# Supplementary material for: Are carbon emissions trading and green financial instruments synergistic? -Comprehensive quantitative research based on content analysis
Source: PLoS One. 2024 Mar 7;19(3):e0298601. doi: 10.1371/journal.pone.0298601 (PMC10919601; doi:10.1371/journal.pone.0298601)
Supplement: S1 Data — (ZIP) [file pone.0298601.s001.zip › Data/地级市Z_F工作报告.zip!/║■─╧/╒┼╝╥╜τ╩╨/└┤╫╘╣1⁄2╓┌║┼í╛┬φ┐╦╩2╛▌═°í┐_2017.docx]

各位代表：

现在，我代表市人民政府向大会报告工作，请予审议，并请各位政协委员和其他列席人士提出意见。

一、过去四年工作回顾侯卫东官场笔记

市六届人大一次会议以来的四年，是张家界发生深刻变化的四年。四年来，我们认真贯彻落实党中央、国务院和省委、省政府决策部署，在市委领导下，团结带领全市各族人民，全面实施“提质张家界打造升级版”战略，真抓实干，攻坚克难，完成了“十二五”主要目标任务，实现了“十三五”良好开局，全面小康建设取得丰硕成果。

过去四年，我们适应经济发展新常态，稳增长、促转型，综合实力稳步提升。面对经济下行的影响和转型升级的任务，我们转变发展理念，突出项目建设，注重转型升级，落实重点部署，实现经济平稳较快增长。地区生产总值由339亿元增长到500亿元（预计数，下同），年均增长8.3%。一般公共预算收入由32.2亿元增长到50.35亿元，年均增长11.9%；城镇、农村居民人均可支配收入分别达到21000元和7800元，与全国、全省平均水平的差距明显缩小。我们充分发挥投资关键作用，实施289个重点项目，固定资产投资由170亿元增加到310亿元，年均增长16.1%；积极培育引导市场消费，社会消费品零售总额由112.7亿元增长到196亿元，年均增长11.6%，消费对经济增长的贡献率由48.8%提升到50.8%。我们坚持以旅游业为主导，着力提高供给质量，重组西线旅游资源，开发创新旅游产品，张家界大峡谷玻璃桥等一批旅游项目建成运营，商务、休闲、户外等旅游业态不断涌现，全域旅游发展格局加快形成。近两年，接待游客规模由3885万人次增长到6143万人次，增长58.1%；旅游总收入由249亿元增长到443亿元，增长77.9%，我市旅游在全省的龙头地位进一步巩固。工业“135”行动计划扎实推进，建设标准厂房86.9万平方米，落户企业118家，张家界经开区规模工业增加值四年增长222%。农业“523”行动计划有效实施，大鲵驯养量达到171.6万尾，发展商品蔬菜基地26.5万亩，农业增加值增长32.5%。服务业发展水平不断提升，引进6家金融机构，设立3支发展基金，直接融资规模扩大到50亿元，金融对发展的支撑作用明显增强；房地产业加快转型，去年中心城区投资和商品住宅销售额分别增长38.8%和43.7%；电子商务、文化体育、健康、物流等产业加快发展，网络经营主体由492户增长到6012户，服务业对经济增长的贡献率达78.3%。去年实施“个转企、小升规”，新增规上企业52家。通过努力，我市主要经济指标增速在全省排位明显提高，旅游接待人次稳居全国同类景区和旅游目的地首位，全社会用电量、固定资产投资、一般公共预算收入增速进入全省前列，显示出良好发展势头，极大增强了全市上下争先创优的信心和决心，为实现“旅游胜地梦全面小康梦”目标奠定了坚实基础。

过去四年，我们致力国际旅游城市建设，提品质、创特色，城市功能不断完善。我们以大交通为重点，建设了张花高速公路，扩建了荷花国际机场，开工了黔张常、张吉怀高铁和张桑高速公路，推进了桑龙、宜张、安慈高速公路等重大项目的前期工作。我们以“三带三区”、“三化三改”为重点，加快中心城区提质步伐。仅用1年3个月时间，就实现了澧水大桥、白马泉高架桥、中心城区15公里滨水环线通车。开工建设了武陵山大道、子午西路延伸段、杨家界大道、沙堤大道等互联快捷通道，新改建119公里城市主次干道、355条小街小巷、63.6公里绿道，推进了邢大等167.3公里旅游干线公路建设，基本建成市民广场。启动了大庸古城、陈家溪特色街区等重点旅游产业项目，推进西溪坪、且住岗、沙堤组团和澧水两岸片区开发，城市社区金融、商业、物业等服务设施不断完善。制定实施棚户区改造5年规划，中心城市近两年就完成了225万平方米的棚改任务。推进交通节点渠化改造，启动10个公共停车场建设，亮化4桥7路105组建筑，拆除36根大型立柱广告和8万多平方米户外广告牌。同时，大力整治交通秩序、渣土扬尘等突出问题，市政保洁水平明显提高，城市管理得到加强，城市面貌不断改观，市民自豪感和满意度不断提升。我们强化规划引领，以国际化旅游城市为目标，加快完善综合交通、市场体系、教育卫生等规划布局，编制城市景观、家具、色彩等专项规划，完善土地利用总体规划、城市规划法定图则体系，实现了中心城市、县城、建制镇控制性规划全覆盖。我们加快县城和中心镇建设，推进慈利、桑植县城扩容提质，建设了洪家关、中湖、三官寺等一批初具规模的特色小镇。全市城镇化率由41%提高到46%，旅游特色新型城镇体系正在形成。

过去四年，我们坚持提高基本公共服务水平，办实事、惠民生，人民群众的获得感不断增强。我们按照“五个一批”，推进“五基工程”、“阳光三保障”和旅游精准扶贫，实施阳光助学4.1万人次、阳光医疗救助3.3万人次，建成阳光房1220套，易地扶贫搬迁9183人，实现20.7万贫困人口脱贫，贫困发生率由22.8%降低至8.9%，武陵源区将率先脱贫摘帽。我们坚持为民办实事，投入150多亿元，建设农村公路1500余公里、保障性住房1.3万余套、养老床位5000余张、义务教育合格学校195所、标准化基层医疗卫生机构382个，解决了34.7万人安全饮水问题，新改扩建了一批城乡农贸市场，启动了城区奇峰市场、农产品批发市场建设。我们着力强化社会保障，实施扩大就业系列政策，开展职业技能培训8.6万人，城镇新增就业4万多人，农村劳动力转移就业38万余人；城乡低保标准不断提高，农村低保实现“应保尽保”，困难群体得到更多关爱；整合城乡居民养老保险、基本医疗保险，实行大病保险等社会保障制度，全市城乡居民的保障水平不断提高。我们大力发展社会事业，加强学校、医疗卫生机构、公共文体设施建设，张家界航院、市高级技工学校实现整体搬迁，张家界一中、市人民医院搬迁工程加快推进，市博物馆建成开馆。深化医药卫生体制改革，取消公立医院药品加成，开展大病医疗救助，实现市级医疗资源“三院合一”。社会事业发展投入增长36.9%。我们始终把安全稳定作为工作底线，强化禁毒工作，打击违法犯罪活动，推进“平安张家界”、“法治张家界”建设和“平安满意在张家界”专项活动，安全生产、食品药品监管、信访维稳、民族宗教、火灾防控等工作进入全省先进行列，综合治理工作蝉联全国社会治安“长安杯”，社会大局保持和谐稳定。

过去四年，我们注重“放管服”改革，破难题、促创新，发展活力不断释放。我们突出行政审批制度改革，完成了10类63项改革任务，推行行政审批事项“两集中两到位”，投资建设项目行政审批时限缩短至48个工作日；深入推进旅游综合改革，建立了“1+3+Ｎ”旅游管理新模式，武陵源核心景区实现区处合一；改革商事登记制度，实行“五证合一”、“一照一码”，市场主体大幅增加；改革张家界经开区管理体制，促进了园区经济发展；改革公交运营体制，树立了城市公交新形象；及时处理一批经济领域遗留问题，化解社会矛盾，维护了良好发展环境。我们正确处理政府与市场的关系，深化要素市场改革，建立公共资源交易中心，推进林权、土地承包经营权确权和不动产登记，要素流动更加有序、便捷；大力发展开放型经济，开通国际货运站、免税店等开放平台，推进外贸“破零倍增”，进出口总额增长91.5%，实际利用内外资分别增长67.1%和91.6%。我们推进大众创业、万众创新，形成了涵盖立项、融资、培训等环节的系列扶持政策，建立了一批众创空间和创业创新孵化基地。近两年，全市新增创业主体9187户，带动就业1.6万余人。专利授权达到1909件，高新技术产业增加值年均增长23.8%。“四新经济”快速发展，成为推动经济增长的强劲引擎。

过去四年，我们落实主体功能区发展布局，重环保、守底线，全市生态文明建设不断加强。我们认识到绿水青山就是金山银山，坚持“在保护中开发、在开发中保护”，扎实开展国家主体功能区建设，万元GDP能耗下降6.45%。我们把环境治理摆在突出位置，实施中心城区雨污分流工程39公里，建成污水管网284公里、污水处理厂6座、大型垃圾处理场4座，污水集中处理能力达到14.5万吨/日，生活垃圾无害化处理量达到26万吨，城市污水处理率达到85%、垃圾无害化处理率达100%，城区澧水河106项检测指标全部达到Ⅱ类标准。深入开展环境整治“6+1”专项行动和农村环境污染综合治理，乡村垃圾收集、转运、处理系统逐步建立，环境卫生明显改善。我们推进生态工程建设，创建国家森林城市，建设澧水河岸护岸林和宝塔岗、樟树角等一批城市公园，中心城区绿化覆盖率由36.3%提高到39.6%。大力实施石漠化治理、水土保持、国土整治等项目，绿化“四沿”810公里，人工造林41万亩，全市森林覆盖率达到70.98%。我们积极开展“两型”单位和空气质量达标城市创建，全市退出烟花爆竹生产企业，中心城区退出烟花爆竹销售企业并实现禁放，建成16个省级生态乡镇、494个市级生态村，武陵源成为省级生态区，我市成为中国绿色旅游示范基地，绿色发展、绿色出行、低碳生活日渐成为人们生产生活的自觉行动。

过去四年，我们坚持不懈加强政府自身建设，深入开展群众路线教育实践活动、“三严三实”专题教育和“两学一做”学习教育；修订完善市政府工作规则，进一步规范投融资、规划、土地管理等重大行政决策机制和程序；自觉接受人大、政协监督，加强行政监察和审计监督，省、市602件人大代表建议和620件政协提案全部办结；严格落实党风廉政建设各项规定，坚决纠正“四风”，形成了团结共事、廉洁干事的良好氛围。

回顾过去四年的工作，我们深刻体会到，做好政府各项工作，必须牢牢抓住发展这个第一要务，致力产业发展，突出项目建设，任何时候都不要动摇；必须坚持深化改革，用改革的办法解决前进中的问题，向前看、算大账、求实效，任何时候都不要迟疑；必须敢于担当、勇于负责，看准了的事果断决策，决定了的事马上落实，部署了的事一抓到底，任何时候都不要退缩；必须让人民群众享受改革发展中的成果，多存惠民情怀、多施惠民政策、多办惠民实事，不断提高人民群众的幸福感和满意度，任何时候都不要懈怠。

回顾过去四年的工作，成绩来之不易，奋斗蕴含艰辛，离不开市委的正确领导，离不开人大和政协的支持与监督，离不开历届政府打下的良好基础，离不开驻张部队、武警官兵的奉献和付出，离不开广大干部群众的齐心协力、艰苦创业。在此，向关心、支持、参与张家界发展的各位代表、委员，同志们、朋友们，表示衷心的感谢和崇高的敬意！

回顾过去四年的工作，我们也清醒地看到，前进中还存在不少困难和挑战：全市经济总量还不大，产业结构不优，旅游综合带动能力有待进一步提升；城市功能不配套，棚户区和城中村改造难度大，旅游城市国际化水平有待进一步提升；供给侧结构性改革任务重，创新发展的动力还不强，开放型经济水平有待进一步提升；群众在养老、教育、医疗、住房、交通等方面仍有许多困难，基本公共服务水平有待进一步提升。我们要直面这些问题，切实增强忧患意识、责任意识，努力在破除瓶颈、扬长补短上取得更多成效。

二、今后五年政府工作思路

市第七次党代会描绘了今后五年全市的发展蓝图。未来五年，我市将全面迈入高铁时代、智能化时代、全域旅游时代，进入脱贫决胜期、投资机遇期、产业转型期。我们要保持定力，增强信心，担当使命，奋勇前行。

今后五年，我们将肩负发展新任务。中央统筹推进“五位一体”总体布局和协调推进“四个全面”战略布局，明确2020年实现农村贫困人口脱贫，贫困县全部摘帽，我们任务艰巨，义不容辞。省第十一次党代会提出建设富饶美丽幸福新湖南，建设经济、科教、文化、生态、开放“五个强省”，经济社会发展整体达到中等发达国家水平，人均地区生产总值力争达到10000美元，我们任重道远，责无旁贷。我市第七次党代会强调，通过五年努力，我市将全面建成小康社会、基本建成国内外知名旅游胜地，我们目标明确，信心百倍。

今后五年，我们将面临发展新机遇。我国进入经济发展新常态，旅游产业作为绿色产业、朝阳产业、幸福产业，在新常态下必将出现快速发展。我们要抓住机遇，深化旅游供给侧结构性改革，加快旅游产业转型升级，发挥旅游综合带动作用，提高旅游产业的规模、质量和效益。我国进入脱贫攻坚决胜期，国家出台一系列重大政策，我们要抓住机遇，加大农村基础设施建设力度，完善基本公共服务体系，壮大农村产业，加快现代农业发展。我国进入新型城镇化快速发展期，我市城镇化水平相对较低，我们要抓住机遇，量力而行完善基础设施，坚定不移保护生态环境，因地制宜发展特色产业，千方百计增加城镇就业，不断提高城镇基本公共服务均等化水平，让人民共享城镇化的成果。

今后五年，我们将融入发展新格局。随着黔张常、张吉怀、宜张、安慈、桑龙等一批重大交通项目建设和航空产业发展，我市将成为大湘西和武陵山片区现代交通枢纽城市，融入区域发展新格局，我们要加快重大道路基础设施建设，实现景区、城区与机场、高铁、高速的互联互通，提高交通便捷性，迎接五湖四海、五大洲四大洋的游客。随着国家“三大区域发展战略”和我省“开放崛起战略”的实施，我市作为国际旅游城市，将融入对外开放新格局，我们要充分发挥一类口岸优势，扩大进出口贸易，努力建成区域进出口基地、优质农产品基地，培育壮大开放型经济，让张家界的商品像旅游品牌一样享誉世界。随着全球旅游经济的增长和全省“锦绣潇湘”全域旅游基地的建设，我市将融入全域旅游新格局，我们要发挥旅游龙头作用，加强国际国内旅游合作，在更大区域推进全域旅游，实现旅游全地域、全领域、全业态发展。

今后五年，我们将培育发展新动能。发展需要动能。我们要注重培育内生动力，探索既要“绿水青山”，又要“金山银山”的绿色发展道路。我们要全面深化改革，突出旅游综合改革、“放管服”改革，完善“三清单一目录”，释放发展动力和活力。我们要鼓励和引导大众创业、万众创新，大力发展非公经济，壮大实体经济，降低经营成本，提高核心竞争力。我们要加强产业融合，挖掘优质特色资源，实现资源充分整合、要素深度融合，提升发展质量和效益。我们要发挥优势，重点发展健康产业，发展会展经济，做强传统旅游，做活生态旅游，做大度假旅游，培育绿色发展新动能。

面对发展新任务、新机遇、新格局、新动能，我们要审时度势，富有远见，以战略眼光，在更长的时期、更大的空间，规划张家界的未来，让张家界走在旅游发展的前列，紧跟时代前进的步伐，开启基本现代化的征程。

今后五年，政府工作的指导思想是：以马克思列宁主义、毛泽东思想、邓小平理论、“三个代表”重要思想、科学发展观为指导，深入学习贯彻习近平总书记系列重要讲话精神，全面贯彻党的十八大和十八届三中、四中、五中、六中全会精神，认真落实省第十一次党代会精神，按照市第七次党代会决策部署，牢固树立新发展理念，坚持改革创新，坚决打赢脱贫攻坚战，夺取全面建成小康社会、基本建成国内外知名旅游胜地新胜利。

经济社会发展的主要预期目标是：经济发展速度高于全省平均水平，居民收入增长速度高于经济增长速度，贫困人口人均收入增长速度高于城乡居民人均收入增长速度。全市经济实力进一步提升，产业结构进一步优化，旅游带动作用进一步发挥，城乡环境进一步改善，人民群众过上更加幸福、更有尊严的生活。实现上述预期目标，政府工作总的要求是：

——坚持落实新发展理念。中央确定的新发展理念，是发展的指南。我们要坚持创新发展，深入实施创新引领战略，推进大众创业、万众创新，促进科技与经济深度融合，推进互联网+和旅游+，培育发展新业态、新产品、新动能。坚持协调发展，正确处理发展中的重大关系，统筹景区、城区、园区融合发展，统筹城乡一体化发展，统筹经济社会协调发展，增强发展整体性。坚持绿色发展，严守环境保护底线和红线，深入开展生态文明和“两型”社会创建，建设宜居宜游宜业张家界。坚持开放发展，统筹国际国内两个市场，扩大对内对外两个开放，提高引资引智引技水平，发展更高层次的开放型经济。坚持共享发展，关注民生需求，办好惠民实事，增进人民福祉，致力让人民群众有更多的获得感，实现更高质量、更有效率、更加公平、更可持续的发展。

——坚持深化供给侧结构性改革。围绕去产能、去库存、去杠杆、降成本、补短板五大重点任务，不断优化供给结构。突出旅游供给侧结构性改革，健全旅游服务体系，提升旅游供给质量，推进全域旅游和旅游国际化，力争接待游客突破1亿人次、旅游总收入突破1千亿元。充分发挥旅游综合带动作用，促进“两型”工业、特色生态农业和现代服务业发展，建设富有旅游特色和较强竞争力的现代产业体系。推进体制机制改革创新，优化产业布局，壮大园区经济，努力把张家界经开区建设成近百亿产值的园区。深化“放管服”改革，优化营商环境，继续推进“个转企、小升规”，培育壮大市场经营主体，不断提高劳动生产率、全要素生产率和潜在增长率。

——坚持建设国际化旅游城市。按照国际化生态文化旅游城市的定位，建设风景优美、文化独特、人人向往的国际化旅游城市。全面提升完善城市功能，加快棚户区和城中村改造，优化综合交通系统，构建布局合理、设施配套、功能完备、安全高效的城市基础设施体系，建设海绵城市、绿色城市、智慧城市、创新城市和人文城市。加快新型城镇化进程，积极培育特色小镇，夯实城镇发展基础，推进农业转移人口市民化。重点支持两个县城的建设和发展，争取慈利撤县设市，实施一批乡改镇、村改社区，辐射带动新农村建设和美丽乡村建设。注重城市文化建设，深刻认识文化始终是国际化旅游城市的不竭资源和永恒魅力，挖掘和弘扬特色文化，促进文化与旅游的融合，提高城市文明程度，不断增强国际化旅游城市的软实力。

——坚持以人民为中心。“人民对美好生活的向往，就是我们的奋斗目标”。要经常感受老百姓的感受，聚焦、聚神、聚力群众合理诉求，补齐基本公共服务短板，提升基本公共服务均等化水平。突出民生基础设施建设，进一步提升水、电、路、通讯等基础设施网络质量。突出扩大就业创业，千方百计增加群众收入。突出发展教育、卫生、文化事业，大力解决群众在教育、医疗等方面的困难。突出完善社会保障制度体系，实现基本养老、基本医疗城乡居民全覆盖。突出精准扶贫精准脱贫，确保在2019年底前，实现现行标准下农村贫困人口脱贫，贫困村全部退出，贫困县全部摘帽，实现全面小康的目标。

各位代表，完成今后五年目标任务，全面建成小康社会，基本建成国内外知名旅游胜地，是全市人民的殷切期盼，是时代赋予的光荣使命！只要我们凝神聚力，苦干实干，做好每年的工作，这个美好蓝图就一定能够实现！

三、2017年主要任务

2017年，是新一届政府履职的开局之年，是实施“十三五”规划承上启下的重要之年，更是深化供给侧结构性改革、推进脱贫攻坚的关键之年。全市上下要全面贯彻党的十八大和十八届三中、四中、五中、六中全会和中央、省委经济工作会议精神，以及省、市党代会精神，紧紧围绕“五位一体”总体布局、“四个全面”战略布局和建设富饶美丽幸福新湖南的部署要求，把握新常态，践行新理念，坚持稳中求进工作总基调，坚持以推进供给侧结构性改革为主线，深入实施“提质张家界打造升级版”战略，稳增长、促改革、调结构、惠民生、防风险，加快推进全面建成小康社会和国内外知名旅游胜地建设，以优异成绩喜迎党的十九大胜利召开。

今年发展的主要奋斗目标是：地区生产总值增长9.5%，规模工业增加值增长9%，固定资产投资增长18%，旅游总收入增长20%，社会消费品零售总额增长12%，一般公共预算收入增长10%，城镇居民和农村居民人均可支配收入分别增长10%和11%。社会治安综合治理、计划生育、安全生产、节能减排等完成省定任务。全面小康实现程度达到90%以上。

实现上述目标，我们要重点做好六个方面工作：

（一）以产业为支撑，不断增强发展后劲。我市经济总量小，根子是产业薄弱，出路在产业发展。必须以更大的决心，更实的措施，推动产业发展。

做大做强旅游产业。旅游是全市的希望，旅游兴则百业旺。必须紧扣现代旅游需求，深化旅游供给侧结构性改革，提高旅游消费和旅游国际化水平，增强旅游综合竞争力。强化旅游营销，继续开发国内市场，大力拓展境外市场，重点巩固韩国和台湾市场、扩大香港和东南亚市场、撬动日本和欧美市场，确保旅游接待人次持续较快增长。丰富旅游产品，加快推进东、西线旅游开发，建设一批旅游项目，发展文化旅游、生态旅游、乡村旅游和健康养生旅游等新型旅游业态。完善旅游服务体系，加快景区基础设施、特色客栈旅馆、休闲度假酒店和户外营地建设，推进旅游“厕所革命”，完善游客中心和“智慧旅游”体系，实现“全域通”网上监管与营运平台的联网运营。加强旅游目的地管理，深化“平安满意在张家界”专项活动，不断提升张家界旅游知名度、美誉度和游客满意度。

突出发展园区经济。保护生态、发展园区经济是我市产业发展的必然选择。必须下大气力加快园区建设，引导产业向园区集中、要素向园区集聚，使旅游综合带动作用得到充分体现。完善园区规划布局，优化功能分区，以智慧旅游、旅游装备、旅游商品、生物医药、食品加工、新型材料等为重点，推进“一区三园”错位发展。加快园区基础设施建设，完善基础设施网络和环保设施，配套基本服务和生活设施，推进标准厂房建设和现代物流配套，提升园区综合服务功能和承载能力。优化园区管理服务，进一步完善园区管理体制，创新投融资机制和建设发展模式，健全创新创业服务平台，构建更加优良的园区发展环境。大力开展园区招商，依托旅游市场和资源优势，引进一批大项目、好项目，确保今年新增入园企业15家以上、园区规模工业增加值增长10％以上。制定实施工业企业成长计划，建立产学研支持体系，推进技术创新和产业链升级，扶持骨干企业做大做强、小微企业加快成长，力争全市新增规模工业企业20家、过亿元工业企业8家。

大力发展特色生态农业。农业是全面小康的基础，是产业发展的重要潜力。必须着眼增加农民收入，深入推进农业供给侧结构性改革，培育农村发展新动能。优化农业产业和产品结构，致力发展绿色优质农产品，做大做强大鲵、蔬菜和茶叶产业，狠抓农产品标准化生产，提升农业特色化、规模化、产业化、品牌化水平。年内大鲵驯养量达到200万尾、商品蔬菜基地达到30万亩、茶园面积达到12万亩，农业增加值增长3.5%以上。推进农田水利建设，以“五小”水利工程为重点，新增和恢复农田有效灌溉面积5.86万亩、高效节水灌溉面积1万亩。创新农业发展机制和模式，实施好“三个百千万”工程，开展好农村土地“三权分置”改革。积极培育发展新型经营主体和服务主体，力争市级以上农业龙头企业达到180家以上。完善科技、金融、农机等现代农业服务体系，推进农村三次产业融合发展，提升农业综合效益和竞争力。

培育壮大现代服务业。服务业发展水平是旅游胜地的重要体现。必须致力创新，加快转型，发展新兴业态，推进现代服务业发展。大力发展电子商务，推进互联网与服务业深度融合，壮大服务业规模，增强服务业的活力和竞争力。大力发展现代金融，引进新的金融机构，完善融资担保体系，推进基金、保险、证券、小贷等金融业态的发展。大力发展新兴服务业，支持设计创意、现代物流、售后服务等产业发展，推动生产性服务业向专业化和价值链高端延伸。以提高品质为导向，引导商贸零售、文化娱乐、养老和社区服务业的健康发展，加快专业市场和大型商贸综合体建设，促进房地产业转型发展，更好满足城乡居民消费需求。

大力发展开放型经济。发挥荷花国际空港优势，主动融入开放大格局，完善通道、通关、平台等开放要素构建，不断提升对外开放水平。突出国际航线网络和货运体系建设，加快实施保税仓项目，争取获批进境指定口岸、生态原产地产品保护示范区和双向免税店，建设张家界旅游航空服务产业基地。继续实施外贸“破零倍增”，培育引进国际贸易经营主体，发展外贸代理和对外劳务合作，努力把我市建设成武陵山片区外贸服务中心。

（二）以项目建设为重点，着力扩大有效投资。落实市第七次党代会决策部署，实现今年预期目标，最关键的是要抓项目，必须通过一个个具体项目，推动全市经济社会加快发展。

推进重点项目建设。深化“项目建设推进年”活动。大力推进大交通项目建设，保障黔张常、张吉怀、张桑等重大项目施工环境，力争桑龙、安慈、宜张等高速公路和安张衡铁路前期工作取得实质性进展。大力推进干线公路、城乡电网、新一代信息网、农田水利、生态环保、地质灾害防御等基础设施项目建设，夯实我市经济社会发展基础。大力推进城市路网、桥梁、管网、堤防等市政基础设施项目建设，加快提升城市综合承载能力。大力推进新型工业和现代农业等产业项目建设，推动产业发展上水平、上台阶。大力推进教育、医疗、科技、文化和体育等民生实事项目建设，不断提高我市基本公共服务水平。

增强招商引资实效。加大招商引资力度，创新招商引资方式，增强工作针对性。强化招商引资基础工作，推进项目库和客户库建设。围绕旅游产业、现代农业、新型工业和现代服务业，注重引进带动性强、附加值高、产业链长的项目。充分利用“港洽周”、湘商大会等重要平台，讲好张家界故事，力争在引进知名企业、知名品牌上取得突破。落实招商引资项目责任制，完善项目履约调度机制，提高招商引资项目签约率、履约率。

强化项目要素保障。进一步拓宽融资渠道，推进金融产品和服务创新，全面激活民间资本，不断提高直接融资比重。加强政银企对接，引导和鼓励金融机构扩大信贷投放。认真把握政策机遇，用好专项基金、融资平台、债券市场、PPP等工具，有效化解项目筹资瓶颈。加大对低效闲置土地的清理处置力度，盘活存量土地资源，推进集约、节约用地。进一步加大征地拆迁力度，保障项目用地需求，进一步提高要素配置效率。

提升项目工作水平。牢固树立项目意识，增强服务主动性。大力优化项目审批程序，落实限时办结、超时问责制度，推行重大项目代办、绿色通道制度。优化经济发展环境，保护投资者合法权益。坚持市级领导联系重点项目制度、部门包干负责制度、重点项目联席会议制度，形成加快项目建设的长效机制。

（三）以人为核心，加快推进新型城镇化进程。按照建设国际化旅游城市的目标，全面提升城市功能、形象和品位，不断满足游客、市民需求，使城市让生活更美好。

推进交通体系建设。加快建设重点道路基础设施，全面推进中心城区滨水环线建设，启动茅溪河、澄潭、枫香岗、红壁岩大桥建设，实现澧水大桥高架桥竣工通车，加快启动磁浮快线等城市轨道交通前期工作。加快推进中心城市和慈利、桑植县城路网配套，建设荷花路、天问路、开明路等21条主次干道，确保沙堤大道、慈利大道、桑植东外环等9条道路建成通车。续建和新建利九、教温、张桃、张慈、三溪、竹黄等25条515公里干线公路，全面改善市域通行状况，为全域旅游发展提供强有力的交通支撑。

增强城市服务功能。以“两供两治”为重点，继续推进城市地下管网改造，实施无事溪、仙人溪截污次干管二期工程，投资建设静脉产业园；改造升级城乡电网和通信网，建设1500个基站，完成且住岗等组团农网改城网任务。加快大庸古城、陈家溪特色街区、张家界国际家居广场、桃花溪谷等项目建设，推进义乌国际小商品城、城乡农贸市场和社区菜市场体系建设。继续实施城市家具提质改造和城市景观的亮化、美化，推进城市公园建设，建设2000个公共停车位、30公里绿道和自行车租赁系统，改造100万平方米以上的城市棚户区，进一步改善居民居住环境，提高城市品位。

加强城市规划管理。遵循旅游城市发展规律，强化城市规划，优化空间布局。按照特色化、国际化的要求，加强对城市空间立体性、平面协调性、风貌整体性、文脉延续性的规划管控，抓好中心城区和武陵源城区整体城市设计。按照“消化存量、杜绝增量”的要求，严格管控违法建设，严格执行城市风貌、色彩、家具等专项规划，全面塑造城市个性和气质。深化规划体制改革，推进国民经济和社会发展规划、城乡规划、土地利用总体规划、生态环境保护规划等“多规合一”，探索建立空间规划“一张图”管理系统。

提升城市治理水平。以人的现代化为核心，优化人居环境，推进城市精细化、全周期、合作性管理。大力解决环境卫生、交通秩序、小区物业管理等突出问题，积极创建全国卫生城市、全国交通管理模范城市、全国环境空气质量达标城市。积极应用现代信息技术治理城市，加快智慧城市建设，推进公安、工商、环保、住建、城管等部门信息互联共享。

做强县城和特色小镇。大力推进慈利、桑植县城扩容提质，支持慈利蒋家坪新区、桑植老观潭新区构建现代城市基础设施体系，加快培育产业带，促进产城更好融合发展。大力提升重点集镇发展水平，改造提质街道、市场、供水等基础设施，培育发展特色产业，着力打造一批支持全域旅游发展的基础平台。

（四）以全面小康为目标，着力办好民生实事。全面小康建设，张家界不能掉队！必须坚持问题导向，突出民生需求，提高全面小康建设的实现程度。

扎实开展精准扶贫精准脱贫。深入实施脱贫攻坚“1332”行动计划，确保完成省里下达的年度减贫任务。进一步完善精准扶贫精准脱贫制度体系，健全识贫、扶贫和防返贫工作机制，动态管理建档立卡贫困对象，规范非贫困村结对帮扶工作，实现贫困村驻村帮扶、贫困户结对帮扶两个全覆盖，确保扶贫资源真正用到贫困对象上，每个贫困群众都能得到有效帮扶。大力推进产业扶贫，鼓励企业联村、大户能人引领、贫困群众以地入股以劳取酬，鼓励贫困户和扶贫企业“分贷统还”使用小额扶贫贷款，鼓励贫困劳动力提升技能、转移就业和自主创业，鼓励贫困乡村利用山、水、生态和传统文化优势，发展乡村旅游、旅游商品生产和旅游后勤服务，不断增强自我发展能力。大力推进易地扶贫搬迁，对接全域旅游、新农村和新型城镇化建设规划，抓好基础设施和产业配套，让贫困群众搬得出、留得住、能致富。大力推进“六到农家”，努力解决跨乡、跨村和较大自然村落的通路、通水、通电、通网络问题。进一步加大阳光助学、阳光医疗救助力度，不让一人因贫失学，不让一户因病、因学致贫和返贫。进一步增强社会保障兜底功能，提高失能家庭低保标准，确保吃、穿不愁，住房、看病、上学有保障。

强化基本民生保障。把解决人民群众饮水安全问题摆在突出位置，全面推进农村饮水安全巩固提升工程，加快农村应急水源水窖和集中供水工程建设，基本解决干旱区饮水问题，确保行政村通自来水率达到80%、农村自来水普及率达到70.5%。着力扩大就业创业，落实大众创业、万众创新系列政策，支持众创空间、创新创业基地加快发展，建立更加有效的就业创业服务体系。年内开展职业技能培训2万人次，发放创业贷款4000万元，城镇新增就业1万人，农村劳动力转移就业1.3万人。织密扎牢社会保障网，坚持“扩面”与“提标”并举，稳步提高城乡低保、特困人员救助、城乡居民基本医疗报销等社保标准，积极推进机关事业单位养老保险制度改革，不断扩大“五大保险”覆盖面。加快“四级社会救助平台”建设，提高养老院服务质量，让留守儿童有美好的明天，让孤寡老人有幸福的晚年，让广大城乡洒满爱的阳光。

协调发展社会事业。坚持教育优先，深化教育综合改革，推进教育优质均衡发展。加强教育基础设施建设，继续抓好城镇学校扩容和合格学校建设，加快中心城区学校改扩新建，完成张家界一中搬迁。大力发展职业教育、民办教育、特殊教育，加快发展学前教育，着力扩大公办和普惠性民办幼儿园覆盖率。下大气力抓好校安工程，为青少年创造良好的学习环境。进一步完善公共卫生服务体系，全面推进医药卫生体制综合改革和公立医院改革试点，加快市人民医院新院、合格乡镇卫生院、社区卫生服务中心和村卫生室建设，推动医疗服务重心下移和诊疗资源下沉。制定实施健康张家界规划，普及应急救护和健康卫生基本常识，努力提升全民健康水平。加强和改进计划生育工作，实施好全面两孩政策，促进人口健康均衡发展。大力发展科技、文化、体育、广电事业，加快建设“一宫四中心”、市科技馆、档案馆和陈家溪体育公园，推进市图书馆、美术馆、大剧院前期工作，全面改变我市公共文体设施长期缺位的局面。加快综合文体站、村文化室建设，争取启动宽带电信普遍服务工程，扩大广播电视无线覆盖面。广泛开展全民健身活动，实施好农民体育健身工程，促进群众体育和竞技体育全面发展。加强国防教育和国防后备力量建设，深入开展双拥共建。促进民族、统计、气象、移民、对台、外事、侨务、档案、地方志、无线电管理等各项事业发展，努力扩大公共产品和公共服务供给，改善和提高人民生活品质。

(五)以法治为遵循，加强和创新社会治理。法治是最可靠的治理。加强和创新社会治理是人民安居乐业、社会安定有序的重要保障。必须坚持专项治理与系统治理、依法治理、综合治理、源头治理相结合，推进政府治理与社会自我调节、居民自治良性互动，构建部门分工履职、全民共建共享的社会治理格局。

着力引导形成良好社会风尚。培育和践行社会主义核心价值观，加强市民文明素质教育，深化群众性精神文明创建，开展文明城市创建活动。深入落实依法治国方略，扎实推进“法治张家界”建设，做好“七五”普法工作，推进人民调解、行政调解、司法调解“三调联动”，营造全社会尊法学法信法守法用法的良好氛围。坚持“法治”与“德治”紧密结合，加强公民道德和诚信建设，完善社会信用体系，使守信者一路畅通、失信者寸步难行。坚持正确舆论导向，以“旅游胜地梦全面小康梦”凝聚共识，汇聚团结奋进的强大力量。

坚守稳定、安全、环保底线。信访是查民情、听民声、解民困的重要渠道。继续推进信访积案攻坚化解活动，切实解决一批历史遗留问题，有效化解社会矛盾，维护群众合法权益。健全公共安全应急体制，完善重大决策社会稳定风险评估机制，有效防范各类突发事件。牢固树立“全面、全程、全员”的大安全观，强化企业主体责任，强化行业主管部门直接监管、安全监管部门综合监管、地方政府属地监管。深入开展安全生产大检查、隐患大排查，杜绝较大及以上安全责任事故，切实保障人民生命财产安全。强化食品、药品、农产品质量安全监管，创建国家食品安全城市，确保游客、市民“舌尖上的安全”。坚持不懈保护生态环境，积极创建国家森林城市，深入推进“两型”社会建设，推广新能源和节材、节地、节水技术，发展装配式住宅。加快实施生态环保重点项目，推进武陵源核心景区移民搬迁，推进城市堤防建设、山洪和地质灾害防治、中小河流和澧水干支流治理，推进澧水、溇水沿岸城镇污水治理，建立河长制，实现河长治。深化环境整治“6+1”专项行动，推行垃圾分类制度，推进畜禽养殖废弃物处理和资源化，实施燃煤全面退城行动，基本淘汰黄标车，不断深化绿色创建，让人民群众在优良的环境中享受美好生活。

全面提升社会治安综合治理水平。着力打造“最平安旅游城市”，推进社会治理体系和治理能力现代化，建设更高水平的“平安张家界”。继续强化突出问题整治，开展“围猎”专项行动，抓好禁毒工作，积极预防、严厉打击各类违法犯罪活动。加强社会治安防控体系建设，推行金融、交通、典当、物流等重点领域实名制，实现“天网工程”和网格化治理村级全覆盖。大力推进社会治安综合治理机制创新，增强公共安全管理服务效能，最大限度地消除治安隐患和治安盲点，实现由被动应对向主动预警预防转变，增强人民群众的安全感。

凝聚共建共享的强大合力。社会治理需要全社会的努力。要注重以法治方式解决宗教领域突出问题，着力构建积极健康的宗教关系。扎实做好基层基础工作，全面加强城乡社区规范化建设，提升基层治理水平。支持工会、共青团、妇联等群团组织依法参与社会治理，切实保障妇女、儿童、残疾人权益。加快行业协会、商会与行政机关脱钩改革，依法规范发展社会组织，推进政府购买社会组织服务，支持专业社会工作、志愿服务和慈善事业发展，调动一切积极因素建设张家界、打造新家园。

（六）以提高执行力和公信力为取向，加强政府自身建设。重任在肩，惟有担当。完成今年各项工作任务，必须坚持党对经济工作的领导，加快转变政府职能，提升施政能力和服务水平，建设人民满意的法治政府、创新政府、廉洁政府和服务型政府。

要解放思想，勇于创新。坚定理想信念，树立“四个意识”，增强“四个自信”，不断提高践行新发展理念的能力和水平。敢于“自我革命”，以锐意创新的勇气、蓬勃向上的朝气，解除不合时宜的思想束缚，破除阻碍发展的条条框框，满怀豪情，激情干事。坚持开阔视野，创新思维，努力走在全省对外开放的前列。

要依法行政，提升效能。坚持法律至上，运用法治思维和法治方式处理解决问题，积极推进市级立法，切实做到严格、规范、公正、文明执法。自觉接受人大及其常委会、人民政协、民主党派和新闻媒体、社会公众等各方面监督，确保权力始终在阳光下运行。深入推进政务公开，深化“放管服”改革，用“更大的放、更好的管、更优的服”推动政府职能转变，努力打造创新创业新洼地。

要勤勉履职，实干担当。坚持主动作为、乘势而为、奋发有为，把加快发展的责任扛在肩上，把群众的冷暖忧乐放在心头，不忘初心，忠诚敬业。坚持良好工作导向，健全绩效考核机制，为担当者担当，为负责者负责，让真心做事的人有机会、有平台、有地位。发扬钉钉子精神，锲而不舍，心无旁鹜，不断把张家界的各项事业推向前进。

要转变作风，廉洁用权。坚持把纪律和规矩挺在前面，严格执行党内政治生活、廉洁自律“两个准则”和纪律处分、党内监督、问责“三个条例”。全面落实党风廉政建设主体责任，以上率下，驰而不息纠正“四风”，努力实现干部清正、政府清廉、政治清明，永葆共产党人政治本色。推进监察体制改革和审计全覆盖。积极预防、坚决惩治腐败，像爱护眼睛一样，维护良好政治生态。

各位代表，回顾过去令人鼓舞，展望未来催人奋进。让我们紧密团结在以习近平同志为核心的党中央周围，在省委、省政府和市委的坚强领导下，同心协力，锐意进取，为夺取全面建成小康社会、基本建成国内外知名旅游胜地新胜利而努力奋斗！
